# Long-Term Irrigation Affects the Dynamics and Activity of the Wheat Rhizosphere Microbiome

**DOI:** 10.3389/fpls.2018.00345

**Published:** 2018-03-21

**Authors:** Dmitri V. Mavrodi, Olga V. Mavrodi, Liam D. H. Elbourne, Sasha Tetu, Robert F. Bonsall, James Parejko, Mingming Yang, Ian T. Paulsen, David M. Weller, Linda S. Thomashow

**Affiliations:** ^1^Department of Biological Sciences, University of Southern Mississippi, Hattiesburg, MS, United States; ^2^Department of Chemistry and Biomolecular Sciences, Macquarie University, Sydney, NSW, Australia; ^3^Department of Plant Pathology, Washington State University, Pullman, WA, United States; ^4^Wheat Health, Genetics and Quality Research Unit, USDA Agricultural Research Service, Pullman, WA, United States

**Keywords:** microbiome, rhizosphere, wheat, soil moisture, *Pseudomonas*, phenazine

## Abstract

The Inland Pacific Northwest (IPNW) encompasses 1. 6 million cropland hectares and is a major wheat-producing area in the western United States. The climate throughout the region is semi-arid, making the availability of water a significant challenge for IPNW agriculture. Much attention has been given to uncovering the effects of water stress on the physiology of wheat and the dynamics of its soilborne diseases. In contrast, the impact of soil moisture on the establishment and activity of microbial communities in the rhizosphere of dryland wheat remains poorly understood. We addressed this gap by conducting a three-year field study involving wheat grown in adjacent irrigated and dryland (rainfed) plots established in Lind, Washington State. We used deep amplicon sequencing of the V4 region of the 16S rRNA to characterize the responses of the wheat rhizosphere microbiome to overhead irrigation. We also characterized the population dynamics and activity of indigenous Phz^+^ rhizobacteria that produce the antibiotic phenazine-1-carboxylic acid (PCA) and contribute to the natural suppression of soilborne pathogens of wheat. Results of the study revealed that irrigation affected the Phz^+^ rhizobacteria adversely, which was evident from the significantly reduced plant colonization frequency, population size and levels of PCA in the field. The observed differences between irrigated and dryland plots were reproducible and amplified over the course of the study, thus identifying soil moisture as a critical abiotic factor that influences the dynamics, and activity of indigenous Phz^+^ communities. The three seasons of irrigation had a slight effect on the overall diversity within the rhizosphere microbiome but led to significant differences in the relative abundances of specific OTUs. In particular, irrigation differentially affected multiple groups of *Bacteroidetes* and *Proteobacteria*, including taxa with known plant growth-promoting activity. Analysis of environmental variables revealed that the separation between irrigated and dryland treatments was due to changes in the water potential (Ψ_m_) and pH. In contrast, the temporal changes in the composition of the rhizosphere microbiome correlated with temperature and precipitation. In summary, our long-term study provides insights into how the availability of water in a semi-arid agroecosystem shapes the belowground wheat microbiome.

## Introduction

Most wheat (*Triticum aestivum* L.) in the Inland Pacific Northwest (IPNW) of the U.S.A. is grown throughout the Columbia Plateau, an area encompassing more than 62,000 square kilometers that comprises the largest contiguous cropping system in the western United States. Cereal crops throughout the region support large populations (10^5^–10^6^ CFU/g of root) of indigenous rhizobacteria that produce the antibiotic phenazine-1-carboxylic acid (PCA) (Mavrodi et al., 2012a; Parejko et al., 2013). Phenazines are colorful, redox-active metabolites that act as electron shuttles (Hernandez et al., 2004; Pham et al., 2008; Wang et al., 2010) and contribute strongly to the morphology, physiology, and ecology of the strains that produce them (Mazzola et al., 1992; Maddula et al., 2006; Price-Whelan et al., 2006; Dietrich et al., 2008). Indigenous populations of PCA-producing (Phz^+^) rhizobacteria from Columbia Plateau soils are diverse and are part of the *Pseudomonas fluorescens* species complex. They include at least 31 genotypes that are closely related to *Pseudomonas synxantha, P. orientalis*, and the provisional species *P. aridus*, and *P. cerealis* (Parejko et al., 2013). These microorganisms are exemplified by *P. synxantha* (formerly *P. fluorescens*) 2-79, a model biocontrol agent isolated from soil at the Washington State University's Lind Dryland Research Station that had been cropped to wheat for 14 consecutive years (Weller and Cook, 1983; Thomashow and Weller, 1988). The production of PCA in strain 2-79 is controlled by the seven-gene *phz* operon that has been cloned, sequenced, and shown to be regulated by quorum sensing (QS) via *N*-(3-hydroxy-hexanoyl)-L-homoserine lactone (Mavrodi et al., 1998; Khan et al., 2005). PCA produced by 2-79-like bacteria has been detected at nanomolar concentrations in the rhizosphere of field-grown wheat, and there is a direct relationship between the amount of phenazines extracted from the roots and the population density of Phz^+^ *Pseudomonas* spp. (Mavrodi et al., 2012a). Phenazines produced by 2-79 and closely related species of Phz^+^ pseudomonads exhibit broad-spectrum antibiotic activity and contribute to the capacity of these organisms to suppress several important plant pathogens (Thomashow and Weller, 1988; Chin-a-Woeng et al., 1998; Arseneault et al., 2016; Jaaffar et al., 2017). Our surveys of commercial wheat fields from throughout the Columbia Plateau revealed that the frequency of root systems colonized by Phz^+^ bacteria is inversely correlated with annual precipitation and irrigation (Mavrodi et al., 2012a,b). This observation prompted us to hypothesize that soil moisture strongly affects the rhizosphere microbiome of dryland wheat and plays a key role in the establishment and proliferation of indigenous phenazine-producing pseudomonads.

The availability of water is a major challenge for agriculture in the IPNW. The region is divided into three annual precipitation zones, of which the low-precipitation zone (<300 mm) encompasses more than 1.6 million cropland hectares. Two-thirds of annual precipitation in the region occurs between October and March, one-fourth during April through June, and <10% in July through September. Across most of the IPNW, winter precipitation is efficiently stored in the soil, underpinning an alternating winter wheat-summer fallow rotation that has been the dominant cropping system for over 125 years (Schillinger and Papendick, 2008). Some parts of the IPNW were considered too dry to farm until the Yakima River Basin and Columbia River Basin projects resulted in the erection of a series of dams and deep wells that capture water for irrigation of over 0.6 million hectares of farmland throughout central Washington and northern Oregon (Schillinger et al., 2010). Much attention has been given to uncovering the effects of soil moisture through management practices on the physiology of wheat grown throughout the Pacific Northwest area. It is also known that the availability of water affects the complex of soilborne diseases of wheat. Take-all, caused by the fungal pathogen *Gaeumannomyces graminis* var. *tritici*, is one of the most important root diseases under irrigation and in higher precipitation areas. Under dryland conditions, take-all is less severe and crown rots caused by *Fusarium culmorum* and *F. pseudograminearum*, and root rots caused by *Rhizoctonia solani* AG-8 and *R. oryzae*, become more important soilborne diseases (Cook and Veseth, 1991). In contrast to the effects of water on cereal crops and soilborne pathogens in the IPNW, much less is known about the role of soil moisture in the establishment, maintenance, and activity of bacterial communities associated with roots of dryland wheat. The missing knowledge is crucial for the rational exploitation of beneficial microbial communities to improve crop performance under conditions of water shortage due to irrigation withdrawal from surface reservoirs and deep wells and shifting rainfall patterns, which are anticipated to become even less consistent as global climate changes (Stockle et al., 2010).

The present work aimed to investigate the effect of water on the population levels, diversity, and composition of microbial communities in the rhizosphere of wheat. The study was conducted by growing wheat for 3 consecutive years in adjacent irrigated and dryland (rainfed) field plots established at the WSU Dryland Research Station in Lind, WA. The experiment was designed to closely mimic conditions in the clusters of irrigated fields scattered across the vast semi-arid wheat-producing area of the Inland Pacific Northwest, US. Our results reveal the magnitude of changes in the wheat rhizosphere microbiome in response to soil moisture, temperature, and crop monoculture. Our findings also shed light on the impact of environmental factors on *in situ* production of a biologically active metabolite in the indigenous community of Phz^+^ rhizobacteria.

## Materials and methods

### Field trial and sampling

Six plots, three irrigated, three non-irrigated, each 18.3 × 18.3 m separated by 3.1 m buffer zones, were established in a completely randomized design at a dryland (never previously irrigated) site at the WSU Lind Dryland Research Station (46.973°N, 118.616°W, 423.7 m above sea level). The soil at the site is a Shano silt loam (coarse-silty, mixed, superactive, mesic Xeric Haplocambids) with uniform texture throughout the profile (39% fine sand, 51% silt, 10% clay), pH 5.63–6.27, and organic matter content of 1.13–1.14%. There is a thin, weak layer of calcium carbonate accumulation at about the 50 cm depth, but otherwise no restrictive layers or rocks within the 180 cm profile. Such soils are typical throughout the low-precipitation zone of east-central Washington (Wuest and Schillinger, 2011). Plots were sown annually from 2011 through 2013 in mid-March with the soft white spring wheat (*T. aestivum* L. cv. Louise). Irrigation (once weekly at night for 12–15 h) began in mid-May from sprinklers installed at the center of the irrigated plots, a regime simulating growers' water application in the same area. Soil matric potential and temperature were monitored with MPS-1 dielectric water potential and ECT temperature sensors and an Em50 data logger (Decagon Devices, Pullman, WA, USA). Sensors were calibrated to equate the relationship between output and soil water potential from −10 to −500 kPa and buried to a depth of 10 and 20 cm. Measurements were recorded hourly during the growing season and average values of water potential and soil temperature per day were calculated and plotted with SigmaPlot (version 10.0; SYSTAT Software, Richmond, CA, USA). Plots were harvested for yield estimation each year in August.

Wheat plants were sampled seven times each year from April through July. Clumps of plants were chosen at random every few meters along each of four perpendicular transects through each plot, dug with a shovel to a depth of about 18 cm, and placed in large plastic bags, four per plot. Plants were brought to the laboratory and stored at 4°C for no more than 24 h before processing. From each bag, four plants were assayed to determine population sizes of total culturable heterotrophic aerobic rhizobacteria, and the bacterial population size and frequency of individual root systems colonized by Phz^+^ pseudomonads containing phenazine-1-carboxylic acid biosynthesis genes.

### Phz+ and total culturable heterotrophic bacteria

Indigenous root-associated Phz^+^ and total culturable heterotrophic bacteria were enumerated by the modified terminal dilution endpoint assay (Mavrodi et al., 2012b). The root system with adhering rhizosphere soil of a single plant was placed in a tube with sterile distilled water (10 or 20 mL), the tube was vortexed (1 min), and then treated in an ultrasonic cleaner (1 min). An aliquot (100 μL) of each root wash was serially diluted in water in a 96-well microtiter plate. The resulting dilutions were then used to inoculate two other microtiter plates pre-filled with: (i) a semi-selective growth medium for fluorescent *Pseudomonas* spp. comprised of one-third-strength King's medium B (^1^/_3_ KMB) (King et al., 1954) supplemented with cycloheximide, chloramphenicol, and ampicillin (100, 15, and 40 μg mL^−1^, respectively); and (ii) medium for growth of total culturable heterotrophic aerobic bacteria consisting of one-tenth-strength Tryptic Soy Broth (^1^/_10_ TSB) (BD Biosciences, Franklin Lakes, NJ, USA) supplemented with cycloheximide (100 μg mL^−1^). Cycloheximide was used to inhibit soilborne fungi, while chloramphenicol and ampicillin were added to reduce the growth of competing soil bacteria because *Pseudomonas* spp. are naturally resistant to both antibiotics (Mavrodi et al., 2007). After incubation at room temperature in the dark for 72 h, the optical density at 600 nm was measured with a Bio-Rad model 680 microplate reader (Bio-Rad Laboratories, Hercules, CA, USA). All wells with detectable bacterial growth (OD_600_ ≥ 0.1) were screened for the presence of Phz^+^ pseudomonads by PCR with the Ps_up1-Ps_low1 primer set targeting the key phenazine biosynthesis gene *phzF* (Mavrodi et al., 2010). Population densities of Phz^+^ pseudomonads were calculated based on the final dilution with positive PCR amplification. All population data were converted to log CFU per gram (fresh weight) of rhizosphere and the detection limit of the bacteria with this assay was log 3.2 CFU g^−1^ root fresh weight. Since not every wheat plant carried Phz^+^ bacteria, the mean population values were reported for colonized plants only. Frequencies of rhizospheres colonized by bacteria were calculated as a proportion of rhizospheres with populations above the limit of detection. The colonization frequency ratio was calculated as a ratio of colonization frequency in non-irrigated over irrigated treatments.

### Extraction of rhizosphere soil DNA and processing of 16S rRNA gene amplicons

**Figure 1:**
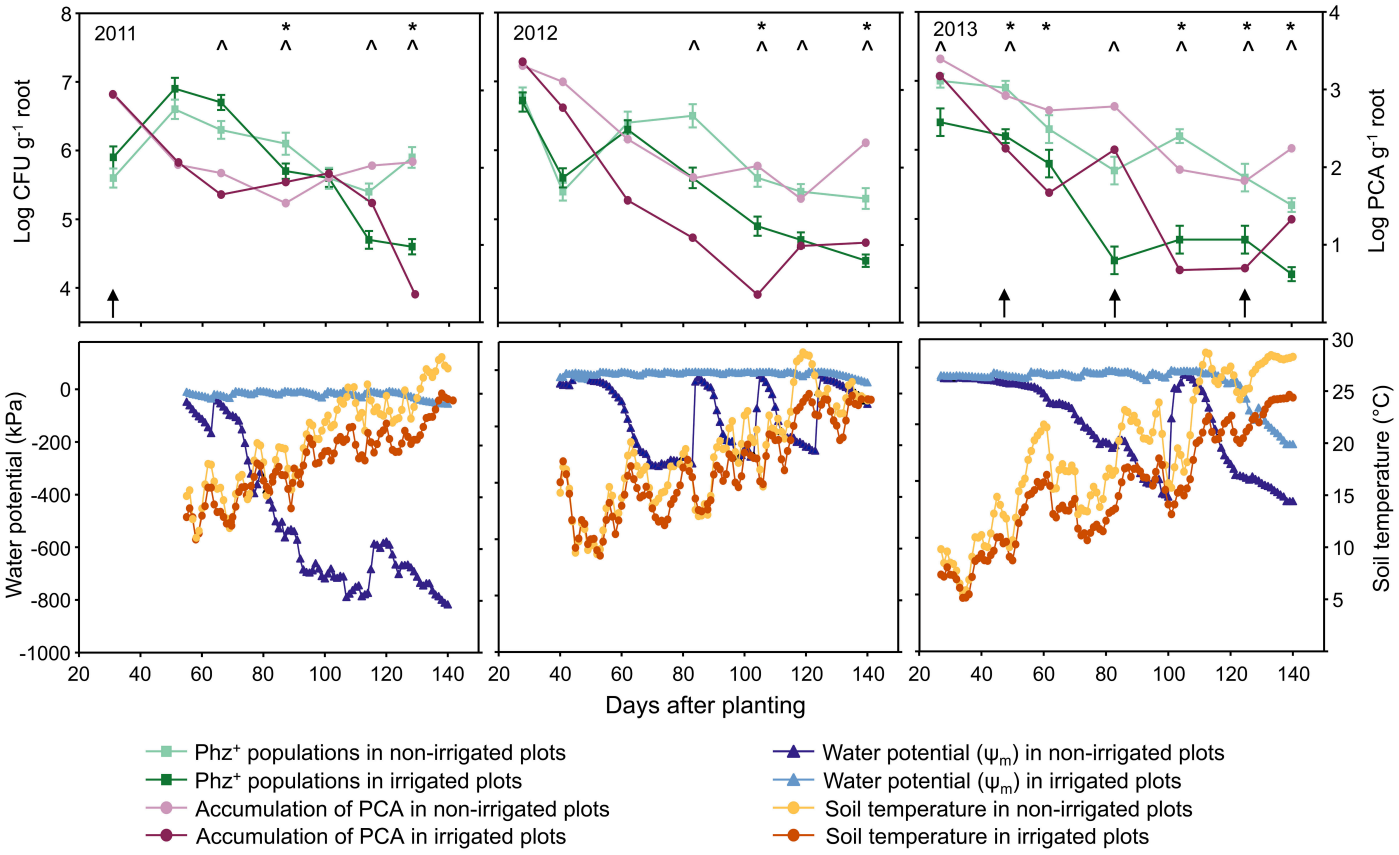
Seasonal dynamics of Phz^+^ *Pseudomonas* spp. and accumulation of PCA on roots of wheat grown in non-irrigated (dryland) and irrigated plots **(Top)**, and changes in the soil water potential (Ψ_m_) and soil temperature at a depth of 10 cm **(Bottom)**. Asterisks and carat signs in the top panel indicate, respectively, sampling points with significant differences in the levels of PCA or Phz^+^ pseudomonads between irrigated and non-irrigated treatments. Vertical black arrows indicate time points when rhizosphere soil DNA was extracted for microbial community analysis.

Rhizosphere soil DNA was extracted from plants collected from each replicate irrigated and non-irrigated plot in the first (Sampling 1) and the third year of the experiment (Samplings 2, 4, and 6; Figure 1). The DNA was purified from root washes of 10 individual plants using an UltraClean Soil DNA Isolation kit (MO BIO Laboratories, Carlsbad, CA, USA) and the alternative protocol for wet soil samples as described by Mavrodi et al. (2007). The quality of the extracted DNA was verified by amplifying 16S rRNA with primers 8F and 1492R (Weisburg et al., 1991). The amplifications were carried out in 25 μL reactions containing 1 × GoTaq DNA polymerase buffer, 200 μM each of dATP, dTTP, dGTP, and dCTP, 1.5 mM MgCl_2_, 20 pmol of each primer, and 0.06 U of GoTaq DNA polymerase (Promega, Madison, WI, USA). The cycling program consisted of the initial denaturation at 94°C for 2 min followed by 30 cycles of 94°C for 20 s, 55°C for 15 s, and 72°C for 1.5 min, with a final extension at 72°C for 3 min. For the microbiome analysis, barcoded 16S rRNA gene amplicons were generated from the extracted rhizosphere DNA by PCR with primers 515F and 806R following the protocol of Caporaso et al. (2010, 2012). The amplifications were performed in 25 μL reaction mixtures containing 5 pmol of each primer and the 5 Prime HotMasterMix (0.5 U HotMaster *Taq* DNA polymerase, 2.5 × HotMaster *Taq* buffer, 2.5 mM MgCl_2_, and 200 μM each of dATP, dTTP, dGTP, and dCTP; Quanta Biosciences, Beverly, MA, USA). Samples were amplified with a T100 gradient thermal cycler (Bio-Rad) and the cycling program consisted of initial denaturation at 94°C for 3 min followed by 27 cycles of 94°C for 45 s, 50°C for 1 min, and 72°C for 1.5 min, a final extension at 72°C for 10 min. The amplicons were purified with a GeneJET PCR Purification kit (Thermo Fisher Scientific, Waltham, MA, USA) and quantified on a Synergy 2 microplate reader (BioTek, Winooski, VT, USA) using a Fluorescent DNA Quantification kit (Bio-Rad). The purified 16S rRNA amplicons were normalized and shipped to AgriLife Genomics & Bioinformatics Services (Texas A&M University, College Station, TX, USA) for sequencing on a HiSeq 2500 instrument (Illumina, San Diego, CA, USA) in 250 bp paired-end rapid mode. Replicates for the Illumina sequencing and subsequent bioinformatics analysis were generated by pooling equal amounts (300 ng) of purified amplicons originating from five randomly chosen wheat plants collected from a single irrigated or non-irrigated plot.

### Microbial community analysis

Individual samples were processed with the FastQC toolkit (http://www.bioinformatics.babraham.ac.uk/projects/fastqc) to confirm sequence quality. Illumina HiSeq forward and reverse reads were merged into contiguous sequences with the mergepairs tool in USEARCH 1.2.22q (Edgar, 2010). The USEARCH fastx_uniques_command was used to dereplicate reads and the UCLUST algorithm was used to cluster reads at a maximum e-value of 0.1 (Edgar, 2010). Operational taxonomic units (OTUs) were characterized by matching to the RDP database (release 11.4; Cole et al., 2014) after clustering with a cutoff of 97% identity. Reads were subsequently mapped back to OTUs to determine OTU abundance for each sample. An OTU was defined as having a minimum of four reads in a cluster (-minsize = 4). The Quantitative Insights Into Microbial Ecology (QIIME) 1.9.1 software suite (Caporaso et al., 2010) was used to calculate the taxonomic tree based on the RDP dataset for use in analyses with the R package phyloseq (Mcmurdie and Holmes, 2013). A hierarchically clustered heatmap was generated with an R (version 3.3.1) batch script (“run_R_heatmap.batch,” provided in Supplementary Material). Any OTUs that accounted for less than 1% of the total OTUs were removed. Phyloseq was used to generate canonical correspondence analysis (CCA) to visualize the community relationships between and within each sample using Bray-Curtis similarity of Log (x+1) transformed values of the abundance of the OTUs. Phyloseq was also used to generate differential abundance plots between the conditions for each sample date using the DESeq2 R package (Anders and Huber, 2010) with *p*-value cutoff of 0.001 and fold change of 2 (log_2_ scale). Alpha diversity was calculated using the vegan package (Oksanen et al., 2013). A Tukey multiple comparisons of means test was performed to determine if there were significant differences in Shannon diversity indices. For specific pseudomonad OTUs, blastn searches were used to look at sequence identity with known pathogenic and biocontrol strains.

### Detection of phenazine-1-carboxylic acid in the rhizosphere of field-grown wheat

Phenazine-1-carboxylic acid was extracted from the rhizosphere of field-grown wheat and quantified as described by Mavrodi et al. (2012a). Briefly, root systems of wheat plants were excised from the shoots and stored in plastic bags at −80°C. Fifteen grams of frozen roots with adhering rhizosphere soil were cut into pieces and shaken for 2 h in 30 mL of 80% acetone acidified to pH 2.0 with 10% trifluoroacetic acid. The extraction efficiency of PCA was determined by spiking each sample with 2 μg of phenazine (Sigma, St. Louis, MO, USA) as an internal standard. The acetone root wash was extracted twice with 10 mL of ethyl acetate and the organic phase was collected, evaporated to dryness, and the dried samples were reconstituted in 1 mL of 98% acetonitrile−2% acetic acid and clarified by passage through a 0.22 μm filter (Bonsall et al., 2007). PCA was detected and quantified with a Waters 2695 liquid chromatograph equipped with a 996 photodiode array and coupled to a quadrupole time-of-flight Q-Tof 2 mass spectrometer with an IonSABRE atmospheric chemical ionization probe (all from Waters Corp., Milford, MA, USA). Samples were separated on a Symmetry C_18_ column (Waters) and spectral scanning by photodiode array was from 180 to 470 nm with monitoring for PCA at 248 nm, its spectral maximum in this solvent system (Mavrodi et al., 2012a). Data were analyzed by using MassLynx, OpenLynx, and QuantLynx software (Waters). PCA was quantified by comparing values to a six-point calibration curve (0.2–10 μg). The determination coefficient (*r*^2^) of the calibration equations ranged from 0.9942 to 0.9997 and the detection limit for PCA in rhizosphere samples was 35 ng per 15 g sample.

### Statistical analyses

Statistical analyses were performed by using appropriate parametric and nonparametric procedures with the Statistix 10 package (Analytical Software, Tallahassee, FL). All population data were converted to log CFU per rhizosphere or gram of root fresh weight. Differences among treatments were determined by the Two-sample *T*-test or Wilcoxon Rank Sum test (*p* ≤ 0.05).

## Results

### The dynamics of rhizobacteria and accumulation of phenazine-1-carboxylic acid in irrigated and dryland wheat plots

The Lind field site is located in the heart of the low-precipitation zone of central Washington State, which is characterized by cool, moist winters and warm, dry summers with an average of 244 mm annual precipitation (http://lindstation.wsu.edu/). In 2011 and 2013, the study site received 198 and 178 mm of precipitation, respectively (Supplementary Table 1). In contrast, the unusually high amount of rainfall in 2012 increased the annual precipitation to a total of 381 mm. For the part of the year when field samples were collected (April through July) the amounts of rainfall in 2011, 2012, and 2013 were 84.6, 137.2, and 63.5 mm, respectively. The mean monthly air temperature during the field sampling period rose from 6.8 to 9.9°C in April to 19.7–23.3°C in July (Supplementary Table 1). In the absence of irrigation, the soil water potential (Ψ_m_) at the depth of 10 cm gradually decreased from −30 kPa in April to −430 kPa in July (Figure 1). With irrigation, the Ψ_m_ values recorded over the field season ranged between −10 and −250 kPa.

**Figure 2:**
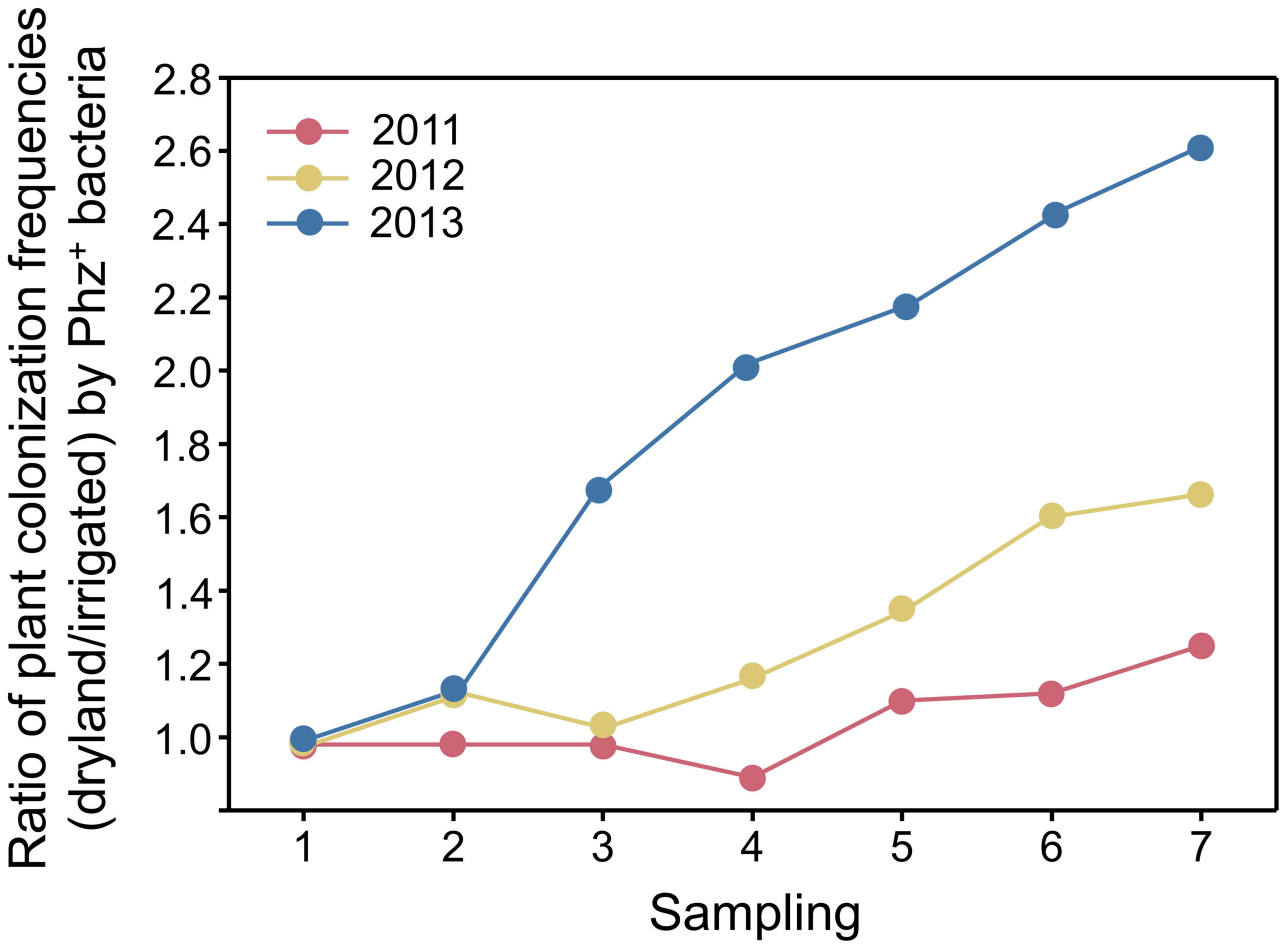
Ratios (dryland/irrigated) of plant colonization frequencies by Phz^+^ pseudomonads in the first, second, and third years of the experiment.

Seasonal changes in temperature and soil moisture were accompanied by fluctuations in populations of total culturable heterotrophic and phenazine-producing rhizobacteria (Figure 1, Table 1). Throughout the study, the counts of culturable rhizosphere bacteria were highest in late April and early May, when they ranged across all treatments between log 7.5 and log 9.2 g^−1^ of root fresh weight. Later in the season, the populations significantly declined, and during the last sampling in July were between log 6.9 and log 7.9 CFU g^−1^ of root fresh weight. There were some statistically significant differences in the levels of rhizobacteria in non-irrigated and irrigated plots, but these variations fluctuated throughout the field season without any apparent trend. The levels of Phz^+^ bacteria exhibited similar seasonal dynamics and peaked (across all treatments) in April and May at between log 5.4 and 7.0 CFU g^−1^ of root fresh weight (corresponds to 0.8–6.3% of the total culturable community). The Phz^+^ populations were lowest in July, when they declined to between log 4.2 and log 5.9 CFU g^−1^ of root fresh weight (0.2–1.3% of the culturable community). Notably, the dynamics of phenazine producers differed significantly in non-irrigated and irrigated plots. There was a significant decline in the number of plants colonized by Phz^+^ rhizobacteria under irrigation, which was especially evident toward the end of the field season (Figure 2). The differences between treatments amplified over time and were highest in July of the third year when 60% of plants were colonized in non-irrigated plots vs. only 23% under irrigation (Table 2). The changes in the plant colonization frequency were accompanied by differences in Phz^+^ population sizes, which declined faster in the irrigated plots (Figure 1, Table 1). On average, at the end of the field season the levels of phenazine producers in the rhizosphere of irrigated wheat were tenfold lower than in the rhizosphere of plants grown without irrigation.

**Table 1:** Levels of indigenous rhizobacteria and Phz^+^ *Pseudomonas* and accumulation of phenazine-1-carboxylic acid (PCA) in the rhizosphere of non-irrigated and irrigated wheat during 2011–2013.

**Sampling date (mm/dd/yy)**	**Treatment**	**Culturable heterotrophic rhizobacteria^*a*^ (log CFU/g root ± S.D.)**	**Phz**^**+**^ **rhizobacteria**		**PCA in the rhizosphere^*a*^ (log per g root ± S.D.)**
			**Population^*a*^ (log CFU/g root ± S.D.)**	**Plant colonization frequency (%)**	
04/18/11	Non-irrigated	8.6 ± 0.4a^*^	5.6 ± 1.0a	96	2.9 ± 0.2a
	Irrigated	8.3 ± 0.6b^*^	5.9 ± 1.1a	98	3.0 ± 0.2a
05/09/11	Non-irrigated	8.6 ± 0.5a^*^	6.6 ± 1.0a	96	2.0 ± 0.3a
	Irrigated	8.7 ± 0.6a^*^	6.9 ± 1.1a	98	2.1 ± 0.7a
05/23/11	Non-irrigated	8.2 ± 0.6a^*^	6.3 ± 0.9b^*^	94	1.9 ± 0.1a^*^
	Irrigated	8.4 ± 0.4a^*^	6.7 ± 0.8a^*^	96	1.7 ± 0.5a^*^
06/13/11	Non-irrigated	7.8 ± 0.6a^*^	6.1 ± 1.0a^*^	85	1.5 ± 0.1b
	Irrigated	8.0 ± 0.4a^*^	5.7 ± 0.8b^*^	96	1.8 ± 0.0a
06/27/11	Non-irrigated	8.3 ± 0.9a	5.6 ± 0.9a	88	1.9 ± 0.2a^*^
	Irrigated	8.4 ± 0.6a	5.6 ± 0.9a	80	1.9 ± 0.0a^*^
07/11/11	Non-irrigated	8.0 ± 0.9a^*^	5.4 ± 0.8a	77	2.0 ± 0.3a
	Irrigated	7.0 ± 0.6b^*^	4.7 ± 0.7b	69	1.5 ± 0.1a
07/25/11	Non-irrigated	7.9 ± 0.4a	5.9 ± 0.9a^*^	75	2.1 ± 0.3a
	Irrigated	6.9 ± 0.4b	4.6 ± 0.6b^*^	60	0.4 ± 0.1b
04/10/12	Non-irrigated	7.5 ± 0.5b	6.8 ± 0.7a	90	3.3 ± 0.1a^*^
	Irrigated	7.9 ± 0.5a	6.7 ± 0.9a	94	3.4 ± 0.2a^*^
04/23/12	Non-irrigated	8.9 ± 0.5b	5.4 ± 0.8a	77	3.1 ± 0.4a
	Irrigated	9.2 ± 0.6a	5.6 ± 0.8a	69	2.8 ± 0.2a
05/14/12	Non-irrigated	8.1 ± 0.4b	6.4 ± 1.0a	79	2.4 ± 0.1a^*^
	Irrigated	8.6 ± 0.5a	6.3 ± 0.9a	77	1.6 ± 1.2a^*^
06/04/12	Non-irrigated	8.1 ± 0.5a	6.5 ± 1.1a	88	1.9 ± 0.3a
	Irrigated	8.2 ± 0.4a	5.6 ± 0.9b	75	1.1 ± 0.8a
06/25/12	Non-irrigated	7.7 ± 0.5a	5.6 ± 0.9a	88	2.0 ± 0.5a
	Irrigated	7.7 ± 0.6a	4.9 ± 0.8b	65	0.4 ± 0.1b
07/09/12	Non-irrigated	7.6 ± 0.4a^*^	5.4 ± 0.7a	81	1.6 ± 0.2a
	Irrigated	7.3 ± 0.5b^*^	4.7 ± 0.5b	52	1.0 ± 0.5a
07/30/12	Non-irrigated	7.0 ± 0.5a	5.3 ± 0.8a^*^	58	2.3 ± 0.1a^*^
	Irrigated	6.9 ± 0.5a	4.4 ± 0.4b^*^	35	1.0 ± 0.6a^*^
04/08/13	Non-irrigated	8.2 ± 0.5b	7.0 ± 0.8a^*^	98	3.4 ± 0.2a
	Irrigated	8.5 ± 0.4a	6.4 ± 1.1b^*^	100	3.2 ± 0.1a
04/29/13	Non-irrigated	8.8 ± 0.6a^*^	6.9 ± 0.9a^*^	96	2.9 ± 0.1a
	Irrigated	9.0 ± 0.4a^*^	6.2 ± 0.7b^*^	85	2.2 ± 0.3b
05/13/13	Non-irrigated	8.0 ± 0.5b	6.3 ± 1.0a	88	2.7 ± 0.1a
	Irrigated	8.3 ± 0.4a	5.8 ± 0.8b	52	1.6 ± 0.0b
06/03/13	Non-irrigated	8.3 ± 0.5a	5.7 ± 0.9a	77	2.8 ± 0.0a^*^
	Irrigated	8.0 ± 0.4b	4.4 ± 0.7b	38	2.2 ± 0.3a^*^
06/24/13	Non-irrigated	8.4 ± 0.6a	6.2 ± 0.8a	100	2.0 ± 0.3a
	Irrigated	8.0 ± 0.5b	4.7 ± 0.9b	46	0.7 ± 0.3b
07/08/13	Non-irrigated	7.3 ± 0.5a	5.6 ± 1.0a	75	1.8 ± 0.3a
	Irrigated	6.9 ± 0.4b	4.7 ± 0.9b	31	0.7 ± 0.3b
07/29/13	Non-Irrigated	7.1 ± 0.6a	5.2 ± 0.8a^*^	60	2.2 ± 0.2a
	Irrigated	6.9 ± 0.5b	4.2 ± 0.2b^*^	23	1.3 ± 0.3b

*Numbers in the same column followed by different letters or different letters with asterisks are significantly different according to two-sample t-test (p = 0.05) or Wilcoxon Rank Sum test (p = 0.05), respectively*.

**Table 2:** Metadata for sampling points at which soil DNA was extracted for microbial community analysis.

**Metadata**	**Treatment**	**Year 1 (04/18/11)**	**Year 3 (04/29/13)**	**Year 3 (06/03/13)**	**Year 3 (07/08/13)**
Water potential (Ψ_m_) at 10 cm (kPa)^*a*^	Non-irrigated	−43	−57	−278	−13
	Irrigated	−36	−32	−16	−48
Water potential (Ψ_m_) at 20 cm (kPa)^*a*^	Non-irrigated	−46	−50	−100	−324
	Irrigated	−45	−40	−26	−110
Soil temperature at 10 cm (°C)^*a*^	Non-irrigated	9.1	12.8	17.3	27.0
	Irrigated	7.1	10.5	13.7	21.9
Soil temperature at 20 cm (°C)^*a*^	Non-irrigated	8.1	12.3	15.9	25.4
	Irrigated	6.5	9.1	13.8	21.8
Phz^+^ population (log CFU/g root ±*S.D*.)^*b*^	Non-irrigated	5.6 ± 1.0a^*c*^	6.9 ± 0.9a^*^	5.7 ± 0.9a	5.6 ± 1.0a
	Irrigated	5.9 ± 1.1a	6.2 ± 0.7a^*^	4.4 ± 0.7b	4.7 ± 0.9b
Plant colonization frequency by Phz^+^ bacteria (%)	Non-irrigated	96	96	77	75
	Irrigated	98	85	38	31
Rhizosphere PCA (log per gram of root ±*S.D*.)^*b*^	Non-irrigated	3.0 ± 0.2a	2.9 ± 0.1a	2.8 ± 0.1a^*^	1.8 ± 0.3a
	Irrigated	2.9 ± 0.2a	2.2 ± 0.3b	2.2 ± 0.3a^*^	0.7 ± 0.3b
Wheat growth stage (Zadok's scale)^*c*^	Non-irrigated	10	13	47	59
	Irrigated	10	13	47	59
Monthly precipitation (mm)^*d*^		25.4	12.7	35.3	0
Air monthly temperature max (°C)^*d*^		13.6	16.7	25.4	33.9
Air monthly temperature min (°C)^*d*^		0.1	1.8	9.3	12.7
Mean monthly air temperature (°C)^*d*^		6.8	9.3	17.4	23.3

a *Water potential (Ψm) and soil temperature at 10 and 20 cm were measured with an Em50 data logger and MPS-1 dielectric water potential and ECT temperature sensors*.

b *Numbers in the same column followed by different letter or different letters with asterisks are significantly different according to two-sample t-test (p = 0.05) or Wilcoxon Rank Sum test (p = 0.05), respectively*.

c *Wheat growth development was assessed by the Zadok's system (10, tillering, emergence; 13, tillering, tillering begins; 47, stem extension, head in the “boot”; 59, heading, head completely emerged)*.

d *Data were obtained from the Northwest Alliance for Computational Science & Engineering (NACSE) database maintained by the Oregon State University (http://www.prism.oregonstate.edu/index.phtml)*.

The amounts of phenazine-1-carboxylic acid recovered from wheat roots closely mirrored the population trend of Phz^+^ rhizobacteria. In both irrigated and non-irrigated plots, the highest amounts of PCA were detected in plants collected in April through mid-May (Figure 1, Table 1). Later in the season, the levels of phenazine-1-carboxylic acid declined and were at their lowest in July. The accumulation of PCA also negatively correlated with soil moisture, and roots of plants collected from irrigated plots during the second or third sampling contained significantly lower amounts of the metabolite (Figure 1).

### Microbial community composition

The profiling of rhizosphere microbiomes was performed using pooled samples of soil DNA extracted from roots of plants collected from replicate irrigated and non-irrigated plots. The control set of DNA samples was collected at the start of the experiment, in April of 2011, and the rest of the samples were collected after three consecutive seasons, in April, June, and July of 2013 (Figure 1, Table 2). Following all quality filtering steps, a dataset of 15,282,474 sequences spanning the V4 region of the 16S rRNA gene from the 2011 controls and the subsequent 2013 samples (*n* = 3 per treatment condition per sampling point, respectively) were compiled (mean length in nucleotides 291, standard deviation 23), all merged reads were truncated to 275 nucleotides after filtering out reads under 275 to provide a uniform read length. After processing, 214 OTUs were detected and after mapping reads back to these OTUs, a total of 9,933,608 reads were utilized in further analyses. OTUs were observed from nine bacterial phyla: *Actinobacteria, Bacteroidetes, Fibrobacteres, Firmicutes, Gemmatimonadetes, Proteobacteria, Synergistetes, Tenericutes*, and *Verrucomicrobia*. The alpha diversity for each sample was calculated and the Shannon diversity measures ranged from 3.7 to 4.6 (Supplementary Figure 1). The Shannon diversity measure for each of the irrigated plots was larger than for equivalent non-irrigated plots, however this difference was not significant based on a Tukey's multiple comparisons of means test.

### Community structure and drivers

**Figure 3:**
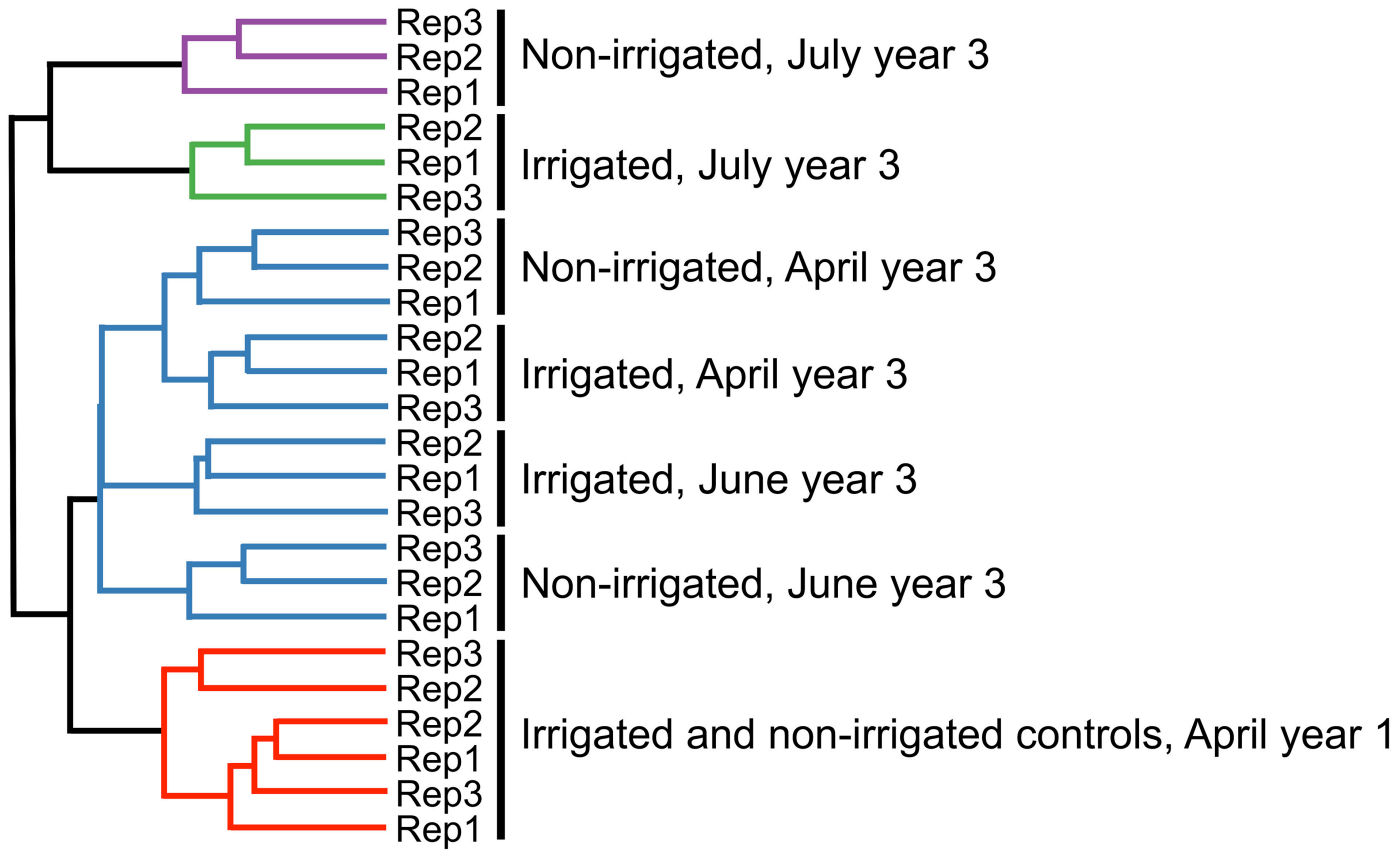
Dendrogram based on Bray-Curtis distance metric hierarchical clustering of OTU relative abundance for each replicate sample.

**Figure 4:**
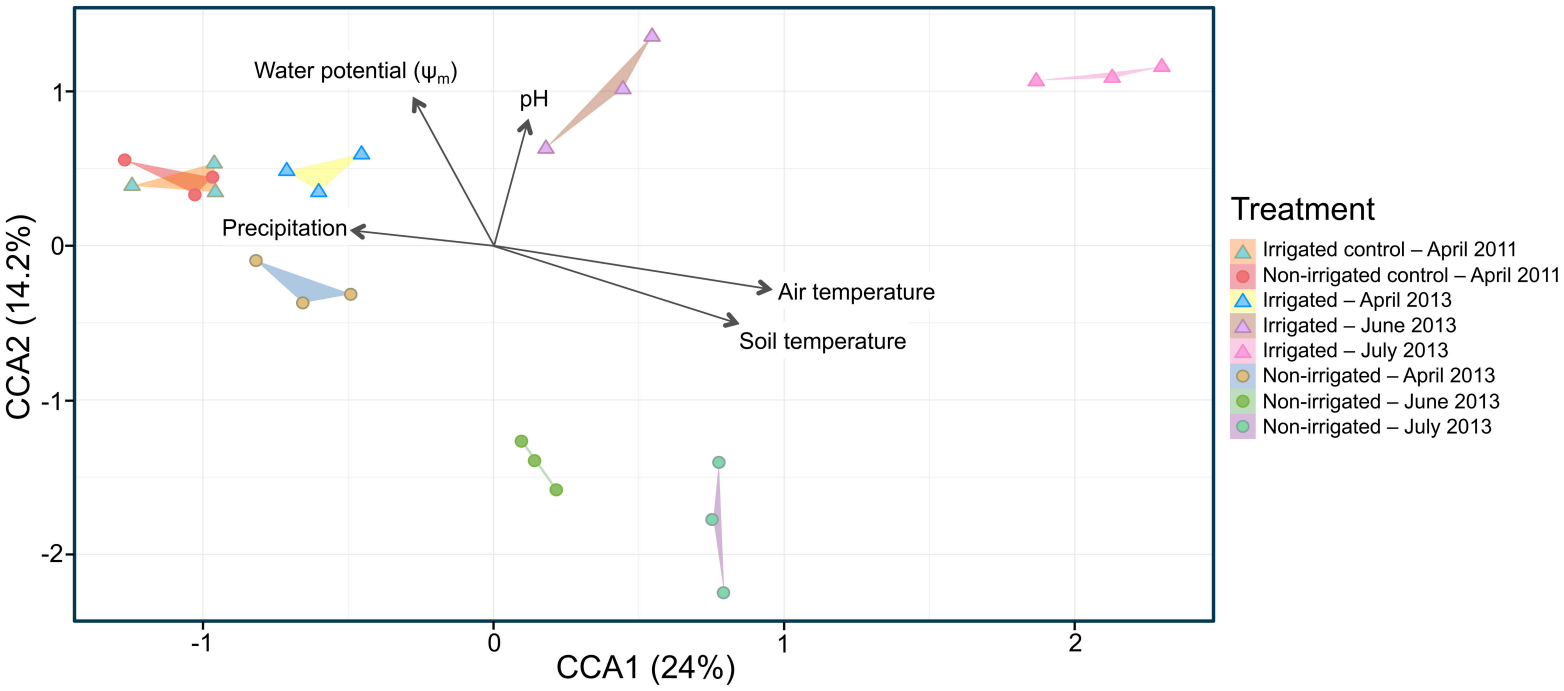
Canonical correspondence analysis (CCA) plot of microbial community composition showing explanatory environmental variables. Each point represents a sampled rhizosphere community. Arrows show quantitative explanatory environmental variables with arrowheads indicating their direction of increase. Samples from irrigated and non-irrigated plots are indicated with triangles and circles, respectively. Colors correspond to control and treatment time points in 2011 and 2013 as shown in the figure legend.

To examine changes in relative abundance of the main OTUs across treatments and time, hierarchical clustering was performed on all OTUs contributing to more than 1% of the observed population (Figure 3). Looking at community composition across the sampling period, we observed increasingly divergent populations between irrigated and non-irrigated treatments. Delineation between the communities from different time points was clearly observed and all replicates were found to cluster with one another (Figure 3). Canonical correspondence analysis was used to determine what environmental variables contribute to the observed shifts in community composition (Figure 4). The total inertia of the plot was 1.0631; of this the constrained inertia was 0.4941. A total of 24% of the constrained inertia was expressed by the CCA1 axis, while CCA2 captured a further 14.2%. The CCA1 axis separates control samples from all treatment samples, with samples collected earlier in the growing season located closer to the controls. CCA1 was strongly correlated with average monthly temperature, soil temperature and, to a lesser degree, precipitation. The separation of samples along CCA1 also reflects changes in the plant age that ranged between the seedling development stage (Zadoks stage13) in late April, through late boot stage (Zadoks 47) in early June to heading stage (Zadoks 59) in mid-July (Table 2). The effect of the growth stage on root microbiome was statistically tied to changes in temperature and moisture (data not shown), and there was no difference in growth stage between plants sampled from non-irrigated and irrigated plots because irrigation commenced later in the field season. Microbial community composition for irrigated samples was well separated from non-irrigated samples along the CCA2 axis and this correlated most strongly with soil water potential and pH.

### Impact of irrigation at the OTU level

**Figure 5:**
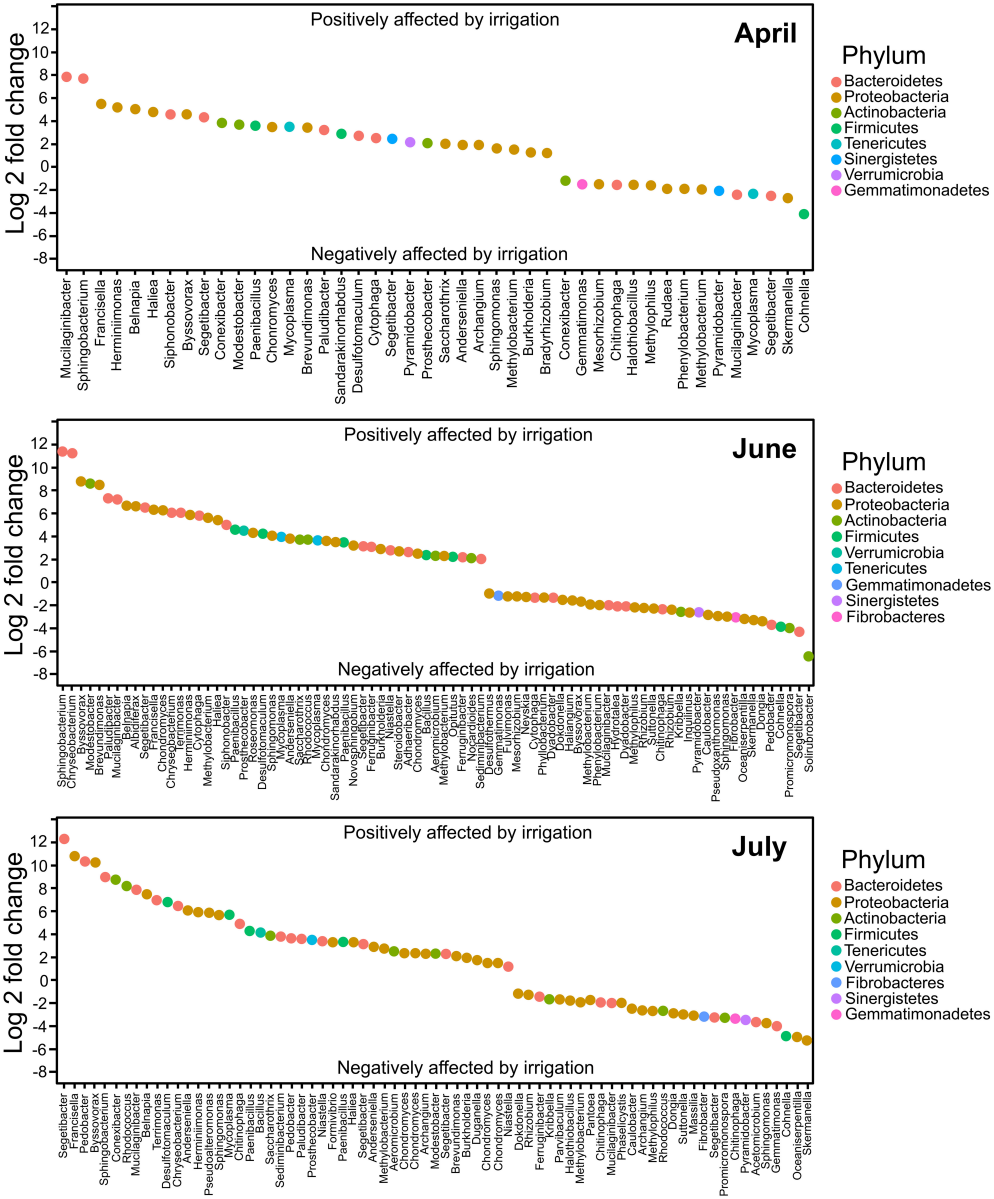
DESeq analysis of taxa differentially distributed in irrigated samples compared to non-irrigated at the three 2013 sampling time points. Analyses were performed with a *p*-value maximum of 0.001. Comparisons were performed with respect to irrigated samples.

Differential abundance analyses were conducted in DESeq2 to determine which taxa were significantly different in non-irrigated compared to irrigated samples at the three 2013 time points used for this experiment. The taxonomic data from 2011 control samples (plots prior to irrigation commencing) were similarly examined using DESeq2. There were 195 OTUs observed in the 2011 control sample sets and there was no significant difference in the relative abundance of these OTUs across sampled control plots. After irrigation commenced, we observed large numbers of OTUs that differed significantly in abundance between irrigated and non-irrigated sites. In April of 2013, 29 OTUs were significantly more abundant in irrigated sites compared to the non-irrigated sites, while 15 were less abundant (*p* < 0.001). In June, this had increased to 47 OTUs significantly more abundant in irrigated sites and 36 less abundant, while the July sample had 44 OTUs significantly more abundant and 29 less abundant in irrigated sites. The degree of abundance differentiation was also higher in the later time points. For the April sample, log2 fold change ranged from 7.8 to −3.9, while in the June and July samples the range has increased, spanning from 11.2 to −6.6, and 12.3 to −5.3, respectively (Figure 5).

For each time point, the five OTUs that showed the highest differential abundance shifts (both up and down) were examined. The OTUs most increased in abundance under irrigation belonged to a relatively small number of phyla (*Bacteroidetes, Proteobacteria, Actinobacteria*; Figure 5, Tables 3, 4). In contrast, representatives of six phyla were observed in the set strongly decreasing in relative abundance under irrigation (*Bacteroidetes, Proteobacteria, Firmicutes, Actinobacteria, Tenericutes, Gemmatimonadetes*; Figure 5, Table 4). Interestingly, at the first sample time point OTU_101, classified to the genus *Mucilaginibacter*, was amongst the most increased under irrigation whilst a second OTU (OTU_58) classified to the same genus was observed to show a strong decrease in abundance under irrigation (Figure 5, Table 3). For OTUs representing pseudomonads, the closest sequence match was determined to ascertain if they were likely soil plant commensals. OTU_4 showed 100% identity to the grass phyllosphere isolate *Pseudomonas cedrina* subsp. *fulgida*, while OTU_10 showed 100% identity to *Pseudomonas syringae* pv. *tomato*, a known plant pathogen. OTU_4 contributed to 4.4% relative abundance in the control sample communities, while OTU_10 contributed to 3.5% relative abundance in the control sample communities. The relative abundance of these OTUs was similar in subsequent sampled communities until the third time point, when the relative abundance of both OTUs declined (Supplementary Figure 2), but was still not significantly different between irrigated and non-irrigated samples.

**Table 3:** OTUs with highest relative increase in abundance under irrigation in April (S1), June (S2), or July (S3) of 2013.

**Sample**	**OTU**	**log2 FC**	**Phylum**	**Class**	**Order**	**Family**	**Genus**	**Species**
S3	OTU_106	12.3	*Bacteroidetes*	*Sphingobacteria*	*Sphingobacteriales*	*Chitinophagaceae*	*Segetibacter*	OTU_106__Segetibacter__*Bacteroidetes*
S2	OTU_179	11.0	*Bacteroidetes*	*Flavobacteria*	*Flavobacteriales*	*Flavobacteriaceae*	*Chryseobacterium*	OTU_179__Chryseobacterium__*Bacteroidetes*
S3	OTU_155	10.3	*Bacteroidetes*	*Sphingobacteria*	*Sphingobacteriales*	*Sphingobacteriaceae*	*Pedobacter*	OTU_155__Pedobacter__*Bacteroidetes*
S2,3	OTU_198	8.6	*Proteobacteria*	*Deltaproteobacteria*	*Myxococcales*	*Polyangiaceae*	*Byssovorax*	OTU_198__Byssovorax__*Proteobacteria*
S2	OTU_116	8.5	*Actinobacteria*	*Actinobacteria*	*Actinomycetales*	*Geodermatophilaceae*	*Modestobacter*	OTU_116__Modestobacter__Actinobacteria
S2	OTU_86	8.3	*Proteobacteria*	*Alphaproteobacteria*	*Caulobacterales*	*Caulobacteraceae*	*Brevundimonas*	OTU_86__Brevundimonas__*Proteobacteria*
S1	OTU_101	7.8	*Bacteroidetes*	*Sphingobacteria*	*Sphingobacteriales*	*Sphingobacteriaceae*	*Mucilaginibacter*	OTU_101__Mucilaginibacter__*Bacteroidetes*
S1,2,3	OTU_97	7.6	*Bacteroidetes*	*Sphingobacteria*	*Sphingobacteriales*	*Sphingobacteriaceae*	*Sphingobacterium*	OTU_97__Sphingobacterium__*Bacteroidetes*
S1,3	OTU_176	5.5	*Proteobacteria*	*Gammaproteobacteria*	*Thiotrichales*	*Francisellaceae*	*Francisella*	OTU_176__Francisella__*Proteobacteria*
S1	OTU_216	5.2	*Proteobacteria*	*Betaproteobacteria*	*Burkholderiales*	*Oxalobacteraceae*	*Herminiimonas*	OTU_216__Herminiimonas__*Proteobacteria*
S1	OTU_109	5.0	*Proteobacteria*	*Alphaproteobacteria*	*Rhodospirillales*	*Acetobacteraceae*	*Belnapia*	OTU_109__Belnapia__*Proteobacteria*

**Table 4:** OTUs with highest relative decrease in abundance under irrigation in April (S1), June (S2), or July (S3) of 2013.

**Sample**	**OTU**	**log2FC**	**Phylum**	**Class**	**Order**	**Family**	**Genus**	**Species**
S2	OTU_64	−6.6	*Actinobacteria*	*Actinobacteria*	*Solirubrobacterales*	*Solirubrobacteraceae*	*Solirubrobacter*	OTU_64__Solirubrobacter__Actinobacteria
S3	OTU_57	−4.8	*Proteobacteria*	*Gammaproteobacteria*	*Oceanospirillales*	*Oceanospirillaceae*	*Oceaniserpentilla*	OTU_57__Oceaniserpentilla__Proteobacteria
S2	OTU_126	−4.5	*Bacteroidetes*	*Sphingobacteria*	*Sphingobacteriales*	*Chitinophagaceae*	*Segetibacter*	OTU_126__Segetibacter__*Bacteroidetes*
S2	OTU_37	−4.2	*Actinobacteria*	*Actinobacteria*	*Actinomycetales*	*Promicromonosporaceae*	*Promicromonospora*	OTU_37__Promicromonospora__Actinobacteria
S1,2,3	OTU_194	−3.9	*Firmicutes*	*Bacilli*	*Bacillales*	*Paenibacillaceae*	*Cohnella*	OTU_194__Cohnella__Firmicutes
S2	OTU_8	−3.8	*Bacteroidetes*	*Sphingobacteria*	*Sphingobacteriales*	*Sphingobacteriaceae*	*Pedobacter*	OTU_8__Pedobacter__*Bacteroidetes*
S3	OTU_189	−3.8	*Gemmatimonadetes*	*Gemmatimonadetes*	*Gemmatimonadales*	*Gemmatimonadaceae*	*Gemmatimonas*	OTU_189__Gemmatimonas__Gemmatimonadetes
S3	OTU_92	−3.6	*Proteobacteria*	*Alphaproteobacteria*	*Sphingomonadales*	*Sphingomonadaceae*	*Sphingomonas*	OTU_92__Sphingomonas__Proteobacteria
S1,3	OTU_28	−2.6	*Proteobacteria*	*Alphaproteobacteria*	*Rhodospirillales*	*Rhodospirillaceae*	*Skermanella*	OTU_28__Skermanella__Proteobacteria
S1	OTU_126	−2.5	*Bacteroidetes*	*Sphingobacteria*	*Sphingobacteriales*	*Chitinophagaceae*	*Segetibacter*	OTU_126__Segetibacter__*Bacteroidetes*
S1	OTU_159	−2.4	*Tenericutes*	*Mollicutes*	*Mycoplasmatales*	*Mycoplasmataceae*	*Mycoplasma*	OTU_159__Mycoplasma__Tenericutes
S1	OTU_58	−2.3	*Bacteroidetes*	*Sphingobacteria*	*Sphingobacteriales*	*Sphingobacteriaceae*	*Mucilaginibacter*	OTU_58__Mucilaginibacter__*Bacteroidetes*

## Discussion

A field survey we conducted in 2008–2009 revealed the presence of large indigenous populations of beneficial phenazine-producing (Phz^+^) *Pseudomonas* spp. in the rhizosphere of wheat grown across 22,000 km^2^ of arid parts of central Washington and northeastern Oregon (Mavrodi et al., 2012a; Parejko et al., 2013). Although these Phz^+^ pseudomonads were ubiquitous and colonized almost 100% of wheat in regions of low precipitation, they were less abundant or non-detectable in irrigated fields or neighboring higher rainfall areas (Mavrodi et al., 2012b). These findings prompted us to hypothesize that precipitation plays an important role in the establishment of indigenous communities and the activity of beneficial Phz^+^ rhizobacteria. In this study, we tested our hypothesis by examining the effect of irrigation on the seasonal dynamics of Phz^+^ pseudomonads and levels of phenazine-1-carboxylic acid in the rhizosphere of field-grown spring wheat. Results of this work strongly supported our hypothesis and revealed that just three successive seasons of overhead irrigation were sufficient to significantly reduce the incidence of Phz^+^ pseudomonads and amounts of PCA in the field (Figures 1, 2). The observed differences between irrigated and non-irrigated plots were reproducible and amplified over the course of the 3-year study, thus identifying precipitation as a key abiotic factor that affects the dynamics and activity of indigenous Phz^+^ *Pseudomonas* communities. Although the mechanism behind this phenomenon is currently unknown, we speculate that increased soil moisture in the irrigated plots perturbs interactions within the rhizosphere microbiome and alters rhizodeposition and soil properties. We attempted to gain additional insights into the effect of irrigation on fluorescent pseudomonads via the sequence-based profiling of the rhizosphere microbiome (see below), but identified only two *Pseudomonas* OTUs that matched the plant pathogen *P. syringae* and *P. cedrina*, which belongs to the *P. fluorescens* group. The levels of these OTUs in the rhizosphere of wheat fluctuated over the duration of the study and did not significantly differ between irrigated and non-irrigated treatments (Supplementary Figure 2). It is likely that differential responses of different *Pseudomonas* to irrigation were obscured by the lack of resolution at finer taxonomic levels due to the relatively short length (300–350 bp) of V4 amplicons used in the microbiome analysis.

Our results also provide the first comprehensive picture of the seasonal dynamics of indigenous Phz^+^ pseudomonads and accumulation of PCA in dryland wheat fields. Over the course of the study, the levels of PCA on wheat roots remained high and peaked in April when the soil was wet and cool and then gradually declined toward the end of the field season. However, the short half-life of PCA in the rhizosphere (Mavrodi et al., 2013) suggests that the production of microbial phenazines on wheat roots is a sustained and highly dynamic process, and continues even as the soil dries to lower matric potential. These findings also indicate that the observed populations of phenazine producers in the field (between 10^5^ and 10^7^ CFU g^−1^ of root fresh weight) were sufficiently high to support HSL-mediated quorum sensing that is necessary for the induction of PCA biosynthesis genes. The sustained production of PCA may play a crucial role in the establishment and maintenance of indigenous communities of Phz^+^ pseudomonads associated with the rhizosphere of dryland wheat. Pseudomonads have respiratory metabolism but can tolerate low oxygen environments such as waterlogged soils. The opportunistic human pathogen *P. aeruginosa* uses redox-active phenazines as alternative terminal electron acceptors, and the PCA-mediated electron shuttling promotes survival of this organism under anaerobic conditions (Wang et al., 2010; Glasser et al., 2014). It is plausible that PCA performs a similar function in rhizosphere Phz^+^ pseudomonads and supports their growth during parts of the year when the soil is wet and hypoxic or even anoxic. Phenazine biosynthesis also modulates the surface adhesion and biofilm architecture in *P. chlororaphis* and *P. aeruginosa* (Maddula et al., 2008; Ramos et al., 2010). In its spatial and temporal characteristics, root colonization by rhizobacteria resembles biofilm growth (Angus and Hirsch, 2013), and PCA may help the Phz^+^ populations to form stress-resistant biofilms and proliferate on roots of dryland wheat growing in soils with intermittent availability of water. Phenazine-1-carboxylic acid has broad-spectrum antimicrobial and antihelmintic properties (Smirnov and Kiprianova, 1990; Cezairliyan et al., 2013) and may aid Phz^+^ pseudomonads in competition with indigenous microflora and resistance to predation by bacteriovorus nematodes. Finally, phenazines play a crucial role in the ability of Phz^+^ rhizobacteria to control soilborne fungal pathogens (Thomashow and Weller, 1988; Chin-a-Woeng et al., 1998; Arseneault et al., 2016; Jaaffar et al., 2017). The combination of higher soil moisture and lower temperatures exacerbates the severity of damage by Rhizoctonia root rot, which is among the most important soilborne diseases of dryland wheat (Smiley and Uddin, 1993; Gill et al., 2001; Smiley et al., 2012). Therefore, the interplay between the precipitation, Phz^+^ populations and the accumulation of rhizosphere PCA early in the spring may have significant implications for managing root and crown diseases of cereal crops in the IPNW soils.

In addition to the effect of irrigation on Phz^+^ rhizobacteria, we examined the impact of altered precipitation on the structure and dynamics of the entire wheat rhizosphere microbiome. Our analysis revealed that roots of dryland wheat harbor diverse communities dominated by *Proteobacteria, Bacteroidetes, Actinobacteria*, and *Acidobacteria*, which is in agreement with earlier assessments of microbial diversity in the rhizosphere of this crop (Yin et al., 2013; Mahoney et al., 2017). The three seasons of irrigation had a slight effect on the overall diversity within the rhizosphere microbiome (Supplementary Figure 1), which was in contrast with the apparent separation of irrigated and dryland treatments by the Bray–Curtis similarity measures that indicated significant differences in the relative abundances of specific OTUs (Figure 3). The observed delineation between rhizosphere populations from irrigated and dryland wheat was particularly pronounced later in the season after the onset of irrigation. The analysis of measured environmental variables contributing to the observed shifts in the community composition revealed that the separation between irrigated and dryland treatments was due to changes in the water potential (Ψ_m_) and pH. In contrast, the temporal changes in the composition of the wheat rhizosphere microbiome were driven by temperature and precipitation (Figure 4). These findings are in agreement with the emerging consensus regarding abiotic factors with marked effects on the variety and abundance of soil microbial taxa (Lauber et al., 2009; Fierer, 2017). The combination of the measured environmental factors explained only part (38.2%) of the total change in the structure of rhizosphere bacterial communities, and the remaining portion of the variation could be due to the complex interaction of other abiotic and biotic factors (e.g., spatial heterogeneity, soil disturbances, dispersal ability, competition, and niche differentiation; Fierer, 2017). The temporal changes in the rhizosphere microbiome are also likely to be driven by plant age and shifts in the composition and amounts of rhizodeposits at distinct stages of plant development. A recent study by Donn et al. (2015) reported a strong effect of the vegetative plant stage on motile, aerobic, or facultatively anaerobic taxa that are tightly associated with wheat roots.

Although water strongly influences soil microorganisms, surprisingly few studies have examined the effect of irrigation on the structure of soil microbial communities across space or time. A recent study by Hartmann et al. (2017) characterized the impact of long-term irrigation on the soil microbiome of a semi-arid pine forest in the Rhone Valley of Switzerland. Results of that study revealed that a decade of irrigation in an ecosystem with a history of water limitation caused a pronounced shift in the microbiome from oligotrophic to more copiotrophic lifestyles. The increased soil moisture and stimulated plant-derived inputs promoted the occurrence of copiotrophic *Proteobacteria* and displaced oligotrophic groups that are more tolerant of water stress such as *Actinobacteria, Gemmatimonadetes, Acidobacteria*, and *Armatimonadetes* (Hartmann et al., 2017). In contrast, our results identified most taxa with strong positive and negative responses to irrigation as *Bacteroidetes* and *Proteobacteria*, and at the class level the largest group of rhizobacteria influenced by irrigation was represented by *Sphingobacteria* (Figure 5; Tables 3, 4). These discrepancies are not surprising and can be attributed to some significant differences between the two experimental systems and sampling procedures. Our study was conducted in an intensively managed agroecosystem dominated by the monoculture of wheat, whereas Hartmann and colleagues studied a natural forest ecosystem located in the European Alps and dominated by Scots pine (*Pinus sylvestris*). Also, it is known that the proximity of plant host strongly influences the composition of soil microbiome (Lareen et al., 2016), and Hartmann and colleagues conducted their analysis by extracting DNA from bulk soil, while we specifically focused on microbial communities that are tightly associated with the surface of wheat roots. Several non-pseudomonad taxa that we identified as differentially responding to irrigation have been previously shown to affect plant health positively. Yin et al. (2013) demonstrated that *Chryseobacterium* and *Pedobacter* from the IPNW wheat field soils produced antifungal compounds and antagonized the mycelial growth of *R. solani* AG-8 under *in vitro* conditions. Furthermore, several isolates of *C. soldanellicola* significantly reduced the severity of Rhizoctonia root rot of wheat in greenhouse assays. A synergistic interaction within a mixture of strains of *Brevundimonas, Pseudomonas*, and *Pedobacter* resulted in a significant growth reduction of phytopathogenic fungi *F. culmorum* and *R. solani* (de Boer et al., 2007). Strains of *Promicromonospora* and *Sphingobacterium* have been found to promote plant growth by secreting gibberellins and modulating the levels of stress-induced ethylene via the production of 1-aminocyclopropane-1-carboxylic acid deaminase (Kang et al., 2012; Feng et al., 2017). Interestingly, although prominently associated with wheat roots, the aforementioned taxa are dynamic and, in addition to irrigation, differentially respond to other agronomical practices such as organic farming, tillage regimens, and the use of different wheat cultivars (Li et al., 2012; Mahoney et al., 2017; Yin et al., 2017).

During the rest of the twenty-first century, all general circulation models predict warmer temperatures and extreme weather events around the globe (Stockle et al., 2010; Zhao and Running, 2010; Seneviratne et al., 2012). As a consequence, crops will increasingly be exposed to abiotic stresses caused by changes in average temperatures, temperature extremes, and moisture availability. Plant roots host distinct bacterial communities that positively influence plant development, vigor, disease resistance, productivity, and response to stressors associated with global climate change (Adriaensen et al., 2005; Rodriguez et al., 2008; Kawasaki et al., 2012; Lau and Lennon, 2012). Much attention has been given to uncovering the mechanisms of water stress in plants. In contrast, the complex effects of altered precipitation on rhizobacteria remain poorly understood. Here, we characterized the responses of biocontrol pseudomonads and the entire wheat rhizosphere microbiome to overhead irrigation. To the best of our knowledge, this is the first study that examined this topic by conducting a long-term field experiment in an agroecosystem with a strong history of water limitation. Our results provide direct experimental evidence that soil water status drives the development of populations of beneficial antibiotic-producing rhizobacteria that contribute to the natural suppression of soilborne diseases of cereal crops. We further demonstrated that these rhizobacteria produce copious amounts of phenazine-1-carboxylic acid that persists in the rhizosphere of wheat over the course of the field season. Phenazines are broad-host-range antimicrobials that inhibit the growth of certain groups of bacteria and fungi (Mavrodi et al., 2006), serve as a source of carbon and energy for others (Costa et al., 2015) and affect the amount and composition of plant rhizodeposition (Phillips et al., 2004), thus raising questions about the broader role of these metabolites in the shaping of the belowground wheat microbiome. More broadly, results of this long-term study provide new insights into how the availability of water in a semi-arid agroecosystem shapes the belowground wheat microbiome. Our findings also suggest that soilborne pathogens in wheat fields across the Inland Pacific Northwest, U.S.A. are kept in check by the concerted action of multiple groups of rhizosphere bacteria, and suggest that the dynamics of these beneficial rhizobacteria may be strongly influenced by the interplay between soil moisture and agricultural management practices. These findings will aid in understanding the impact of agricultural management practices and climate change on the dynamics of the rhizosphere microbiome and soilborne pathogens in cereal crops.

## Author contributions

DM, LT, DW, and IP: conceived the research project; OM, DM, JP, and MY: collected field samples; OM: generated amplicons for Illumina sequencing; RB: extracted and quantified phenazine-1-carboxylic acid; LE and ST: performed the microbiome analysis; DM, OM, LE, ST, DW, and LT: wrote the manuscript and all authors contributed to the manuscript revision.

### Conflict of interest statement

The authors declare that the research was conducted in the absence of any commercial or financial relationships that could be construed as a potential conflict of interest.
