# Supplementary material for: Long-Term Irrigation Affects the Dynamics and Activity of the Wheat Rhizosphere Microbiome
Source: Front Plant Sci. 2018 Mar 21;9:345. doi: 10.3389/fpls.2018.00345 (PMC5871930; doi:10.3389/fpls.2018.00345)
Supplement: Supplementary file 1 [file Image1.PDF]

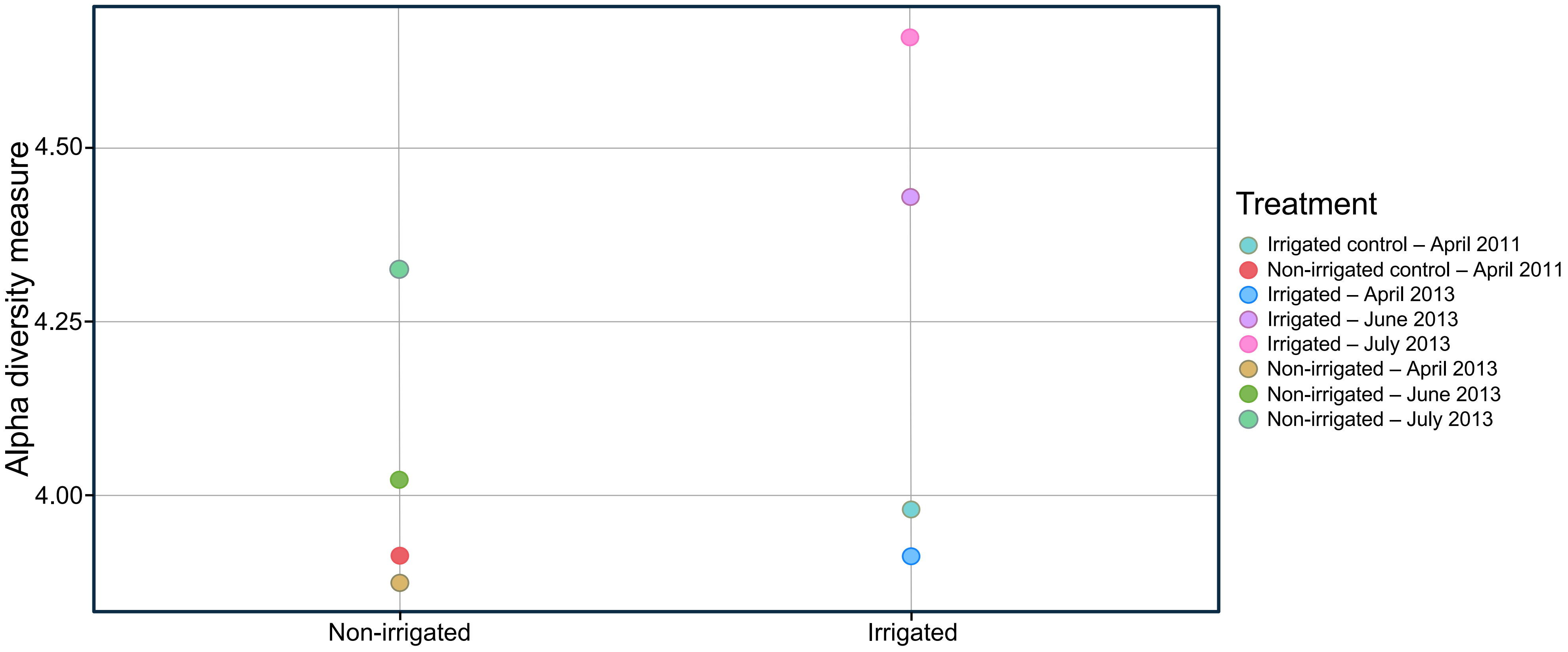

Supplementary Figure 1. Shannon alpha diversity measures for all samples. The values presented are the averages for each set of replicates.
