# Supplementary material for: Long-Term Irrigation Affects the Dynamics and Activity of the Wheat Rhizosphere Microbiome
Source: Front Plant Sci. 2018 Mar 21;9:345. doi: 10.3389/fpls.2018.00345 (PMC5871930; doi:10.3389/fpls.2018.00345)
Supplement: Supplementary file 2 [file Image2.PDF]

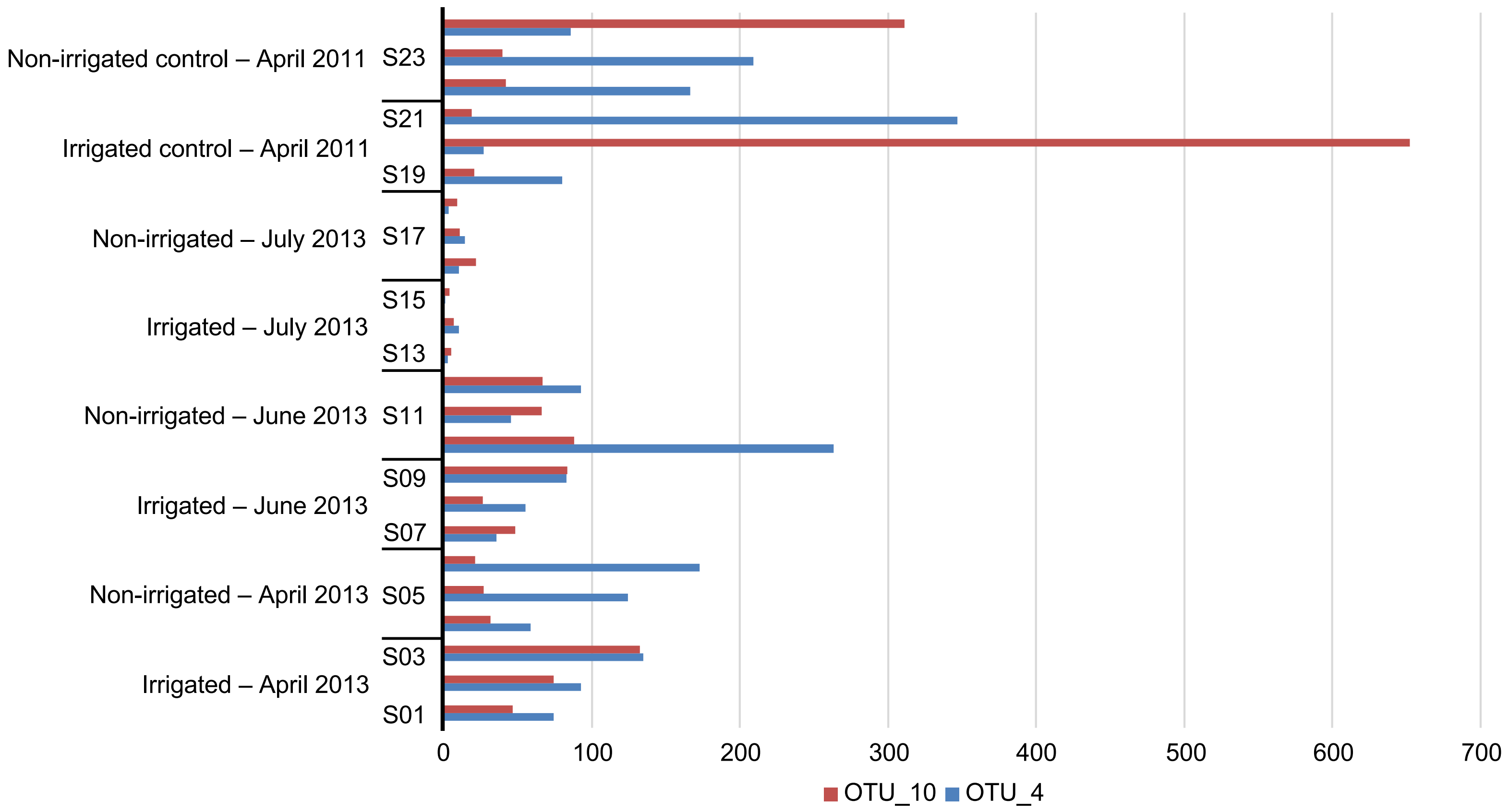

Supplementary Figure 2. Comparison of relative abundance of reads classified as OTU_4 and OTU_10. The raw read numbers were normalized against total read counts and multiplied by 10,000 to produce a whole number scale.
