# Supplementary material for: Long-Term Irrigation Affects the Dynamics and Activity of the Wheat Rhizosphere Microbiome
Source: Front Plant Sci. 2018 Mar 21;9:345. doi: 10.3389/fpls.2018.00345 (PMC5871930; doi:10.3389/fpls.2018.00345)
Supplement: Supplementary file 3 [file Table1.DOCX]

Supplementary Table 1. Monthly precipitation and mean air temperature recorded at Lind Dryland Research Station in 2011, 2012, and 2013.

| Month | Precipitation (mm) | | | Mean temperature (°C) | | |
| --- | --- | --- | --- | --- | --- | --- |
|  | 2011 | 2012 | 2013 | 2011 | 2012 | 2013 |
| January | 25.7 | 20.3 | 18.8 | -0.3 | 1.0 | -2.8 |
| February | 6.4 | 22.4 | 6.1 | 0.2 | 2.7 | 3.1 |
| March | 34.5 | 50.8 | 14.2 | 5.5 | 5.2 | 6.3 |
| April | 25.4 | 32.8 | 12.7 | 6.8 | 9.9 | 9.3 |
| May | 39.9 | 8.4 | 15.5 | 11.5 | 12.6 | 13.9 |
| June | 10.7 | 58.9 | 35.3 | 15.8 | 16.3 | 17.4 |
| July | 8.6 | 37.1 | 0 | 19.7 | 22.9 | 23.3 |
| August | 0 | 1.5 | 26.9 | 21.7 | 22.2 | 22.6 |
| September | 2.0 | 0 | 21.6 | 20.1 | 17.2 | 19.1 |
| October | 20.6 | 37.8 | 1.27 | 12.1 | 9.9 | 9.5 |
| November | 10.4 | 64.0 | 16.8 | 3.0 | 6.4 | 2.6 |
| December | 13.2 | 48.5 | 9.4 | 2.1 | 2.8 | 3.3 |

*^a^* Data were obtained from the Northwest Alliance for Computational Science & Engineering (NACSE) database maintained by the Oregon State University (<http://www.prism.oregonstate.edu/index.phtml>).
